# Development of a Comprehensive Radiation Oncology Quality and Safety Program

**DOI:** 10.3389/fonc.2014.00030

**Published:** 2014-02-20

**Authors:** Edward S. Sternick

**Affiliations:** ^1^Rhode Island Hospital, Warren Alpert Medical School of Brown University, Providence, RI, USA

**Keywords:** radiotherapy, quality, safety, ionizing radiation, medical physics

Radiation oncology practice is a collaborative effort that involves multiple clinical and technical specialists including physicians, medical physicists, dosimetrists, radiation therapy technologists, and nurses. The safe and efficient delivery of radiation therapy was never a simple matter and is now exceedingly complex.

The growing complexity of radiation oncology procedures has been identified as a significant risk factor in potentially causing patient treatment errors. National and international standards have evolved to guide the safe and effective use of ionizing radiation for treatment and have been codified by leading professional organizations into accreditation requirements that necessitate rigorous documentation of performance for each aspect of the process of care. The process of care in radiation oncology refers to a conceptual framework for guaranteeing the appropriateness, quality, and safety of all patients treated with radiation for cancer and certain benign conditions.

A primary objective of a Comprehensive Radiation Oncology Quality and Safety Program is the design and clinical implementation of approaches that will minimize the incidence of radiotherapy adverse events that could potentially have negative impacts on the quality of patient care.

The papers in this Research Topic address multiple issues that should be considered to ensure managing the process of care with approaches that will ensure the optimal delivery of radiation therapy.

